# A cadaveric study of the posterior band of the inferior glenohumeral ligament of the shoulder and its dynamic behaviour in different arm positions

**DOI:** 10.1002/jeo2.12014

**Published:** 2024-03-09

**Authors:** Geoffroy Nourissat, Claire Bastard, Claire Cammas, Vincent Salabi, Anselme Billaud

**Affiliations:** ^1^ Clinique de l'Épaule—Paris—Clinique Maussins‐Nollet Paris France; ^2^ Hôpital Saint Antoine Service de Chirurgie Orthopédique Paris France; ^3^ CHU de Fort de France Guadeloupe France; ^4^ Clinique Bouchard Centre Main Épaule Méditerranée Marseille France; ^5^ Clinique du Sport de Bordeaux Mérignac Mérignac France

**Keywords:** Bankart, IGHL, labrum, posterior band, shoulder instability

## Abstract

**Purpose:**

The inferior glenohumeral ligament (IGHL) is composed of three parts: the anterior branch or band (AB), the axillary pouch and the posterior band (PB). The latter has rarely been studied. We aim to describe the PB of the IGHL and its dynamic behaviour in different arm positions.

**Methods:**

Twelve fresh cadaveric shoulders were used and the two bands (AB and PB) of the IGHL were dissected and isolated, taking away all muscle, ligaments and capsule. Characteristics of the bands were studied in five positions: maximum external rotation (ER1), abduction (ABD), internal rotation (IR), ABD external rotation (ER2) and anterior elevation–adduction–IR (Hawkins–Kennedy test position). Progressive and randomized sectioning of the bands and capsule with a scalpel was performed to study its impact on mobility and translation of the glenohumeral joint.

**Results:**

The bands that tensioned first were in ER1, the AB at 97 ± 9° (80–110); in ER2, the AB at 81 ± 19° (30–100); in IR, the PB at 64 ± 9° (50–80); and in ABD, the PB at 87 ± 10° (70–105). Isolated sectioning of the AB had no effect on ABD, whilst isolated sectioning of the PB allowed greater ABD. In ER2, the AB limited anterior translation. After sectioning the AB, anterior translation remained limited by the PB, which wrapped around the humeral head and locked the joint by pressing the two joint surfaces tightly together. In Hawkins–Kennedy position anterior elevation–adduction–IR, the AB is the first constraint and the posterior translation was limited by the PB alone only in four cases.

**Conclusions:**

When the IGHL is isolated, ligament limitation of glenohumeral ABD seems to be uniquely dependent on the PB. In the Hawkins and Kennedy position, the AB is the first constraint. In the case of an isolated lesion to the AB, the PB participates in anterior stabilization of the shoulder by wrapping around the humeral head that cannot dislocate. These findings confirm the role of the PB in glenohumeral joint stability.

**Level of Evidence:**

Level IV.

**Abbreviations**
AB: anterior band
ABD: abduction
ER1: maximum external rotation
ER2: abduction external rotation
IGHL: inferior glenohumeral ligament
IR: internal rotation
PB: posterior band

## INTRODUCTION

The glenohumeral joint is an extremely mobile joint that is stabilized by muscles and ligaments. Three ligaments cover the capsule: the superior glenohumeral, the middle glenohumeral and the inferior glenohumeral ligament (IGHL) [1]. The IGHL is composed of three parts: the anterior band or branch (AB), the posterior band (PB) and the junction between the two bands, which forms the axillary pouch. A number of studies have described the insertion of the AB into the IGHL, but no study to date has described the PB [5, 6, 9, 10, 12] in different shoulder positions. The anatomy of the PB, particularly its humeral and glenoid insertion, its insertion in relation to the inferior margin of the glenoid cavity and the axillary pouch or posterior capsule have been studied, but its dynamic movements during movement of the shoulder, notably in the dislocated position, has not been studied, which limits our knowledge on its functional role in a mild or severe Bankart lesion [5]. Thus, the benefit of repairing the PB in cases of dislocation is unknown. Therefore, the purpose of our study was to carry out a descriptive anatomical evaluation of the PB of the IGHL. Our hypothesis is that the dynamic behaviour of the PB of the IGHL changes in different shoulder positions effecting glenohumeral stability.

## METHODS

Twelve fresh–frozen cadaveric shoulders free from arthritis or previous surgery were used in the evaluation of this study (eight male [67%], six right shoulders [50%], mean age 74 years old [64–78]). Specimens were collected at the fer à Moulin anatomy laboratory of APHP in Paris. The deltoid, clavicle, pectoralis major and complete rotator cuff were removed conserving only the inferior, posterior and superior capsule and the inferior, anterior and posterior GHL and its axillary pouch. The humerus and scapula were conserved in their entirety and were placed on a support jaw allowing reproducible orientation of the glenoid cavity of each shoulder, both perpendicular and horizontal with regard to the surface and profile. The plane of the glenoid cavity was defined as the reference plane for all movements performed. The AB of the IGHL was systematically identified and measured. The length of its proximal and distal insertion zone and the distance of its insertion proximal from the labrum and distal from the humeral head were measured with a caliper with a nominal precision of 1 mm. The localization of its insertions was evaluated using the ‘hours of the clock’ with an insertion between 0 and 6 o'clock for the anterior part of the glenoid cavity and from 0 to 6 o'clock to the humerus. The insertion in the glenoid cavity, which was either direct or medial, was measured in millimetres in relation to the labrum. Its average thickness in the middle part was measured and it was systematically noted whether it was possible to clearly differentiate it from the capsule and in its minimum tension position. As soon as the ligament was fully under tension, measurements were done. They were performed in 0° of abduction (ABD) or rotation, by pulling solely, laterally on the humeral head opening of the glenohumeral joint.

The same values were measured for the PB, after identifying its proximal insertion in relation to the labrum and distal in relation to the humeral head, using the values from 6 to 12 o'clock.

The positions of both the AB and the PB of the IGHL were evaluated visually in five different positions:

‐ *Maximum external rotation (ER1)*: Axis of the humerus perpendicular to the axis of the glenoid cavity at 0° ABD.
‐ *Maximum internal rotation (IR)*: Axis of the humerus perpendicular to the axis of the glenoid cavity at 0° ABD.
‐ *ABD*: Maximum abduction at 0° external rotation corresponding to the Gagey (hyperabduction) test [3, 4].
‐ *ABD to 90° and maximum external rotation (ER2)* [2, 3].
‐ *Anterior elevation to 90°, adduction 30° and IR*: Corresponding to the Hawkins–Kennedy test, as well as the Kim test when clinically diagnosing for posterior glenohumeral instability [6, 7].

The plane of the glenoid cavity was used as the reference to measure the glenohumeral positions, the superior–inferior vertical axis corresponding to the axis passing through 0 and 6 o' clock position, the horizontal from 3 to 9 o'clock. Translation, adduction–ABD, internal and external rotation were defined as a function of the humeral–epicondyle axis in relation to the plane of the glenoid cavity. The position of the glenohumeral joint to identify the maximum tension for both the AB and PB of the IGHL was recorded. This was defined as the position where the ligament had maximum extension. Beyond this position the GHL bands did not stretch any further. All specimens were mounted on a special designed vice clamp and the forces to the shoulder were applied manually until the ligament was completely taut.

These same positions of the shoulder were then performed on six shoulders after isolated sectioning of the PB of the IGHL. Shoulder motion and translation were then measured and recorded visually.

## RESULTS

### Anatomical description

**Figure 1:**
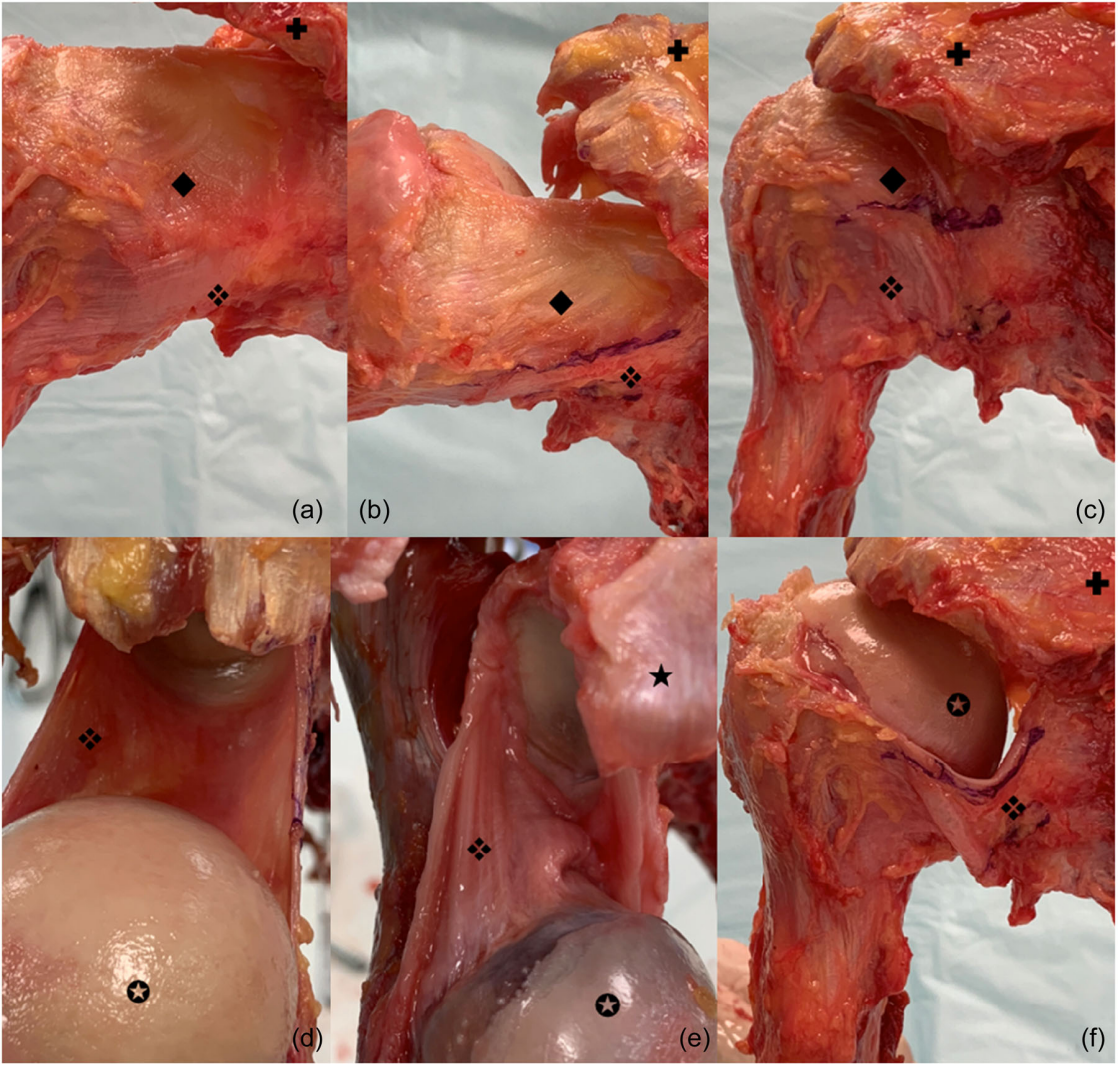
Posterior aspect of the shoulder. ❖, posterior band of the inferior glenohumeral ligament (IGHL); ◆, intermediate capsular zone; ✪, humeral head; ✚, acromion; ★, coracoid process. (a) The posterior band of the IGHL is located under the posterior intermediate capsular zone. (b) The difference between capsule and posterior band is clearly identified. (c) Thickening of the posterior capsule defines the limit between the posterior intermediate capsular zone and the posterior band. (d) Superior view of the IGHL with posterior band. Proximal insertion is located medial to the labrum on each side of the glenoid. Distal insertion is far from the cartilage of the humeral head. (e) Posterior band of the IGHL is located medial to the labrum and distal insertion is far from the cartilage of the humeral head. (f) Superior view of the posterior band of the IGHL after removal of the posterior intermediate capsular zone.

The mean (SD) length and width of the AB were 47 ± 6 mm (range: 40–52) and 14 ± 3 mm (10–20), respectively, and its mean thickness was 1.8 ± 0 mm (1.4–2.0). The proximal insertion of the AB was from 3 to 5 o'clock for the right shoulders and from 7 to 9 o'clock for the left shoulders (Figure 1a,b). The latter was inserted on the labrum in 1/12 cases, but on average 0.9 ± 0 mm (0.4–1.2) beyond the labrum. The distal insertion was from 7 to 9 o'clock for the right shoulders and 3 to 5 o'clock for the left shoulders. It was always distant from the cartilage.

Concerning the PB, mean length and width were 54 ± 7 (50–60) and 16 ± 5 mm (10–25), respectively. Mean thickness was 1 ± 0 mm (0.2–1) when it could be differentiated from the capsule (*n* = 10/12) (Figure 1a,b). The proximal insertion of the PB was from 6 to 9 o'clock for the right shoulders and from 3 to 6 o'clock for the left shoulders, and the distal insertion was from 3 to 6 o'clock for the right shoulders and from 7 to 9 o'clock for the left shoulders. It was inserted on the labrum in 2 of the 12 cases, but on average 1 ± 1 mm (0.5–1.2) past the labrum (Figure 1c–f).

### Positional description

In maximal external rotation, the AB is most taut at an average of 97 ± 9° (80–110). After sectioning the AB, the PB did not become taut. In IR, the PB tensioned at 64 ± 9° (50–80) but the AB never became taut.

In ABD, the PB became tensioned at 87 ± 10° (70–105). If ABD was continued beyond this, the PB forced external rotation to continue ABD without systematically tensioning the AB. The latter was tensioned in 4 of the 12 cases at 97 ± 43° (60–160). Beyond this point, PB tension forced external rotation to ABD external rotation (ER2).

**Figure 2:**
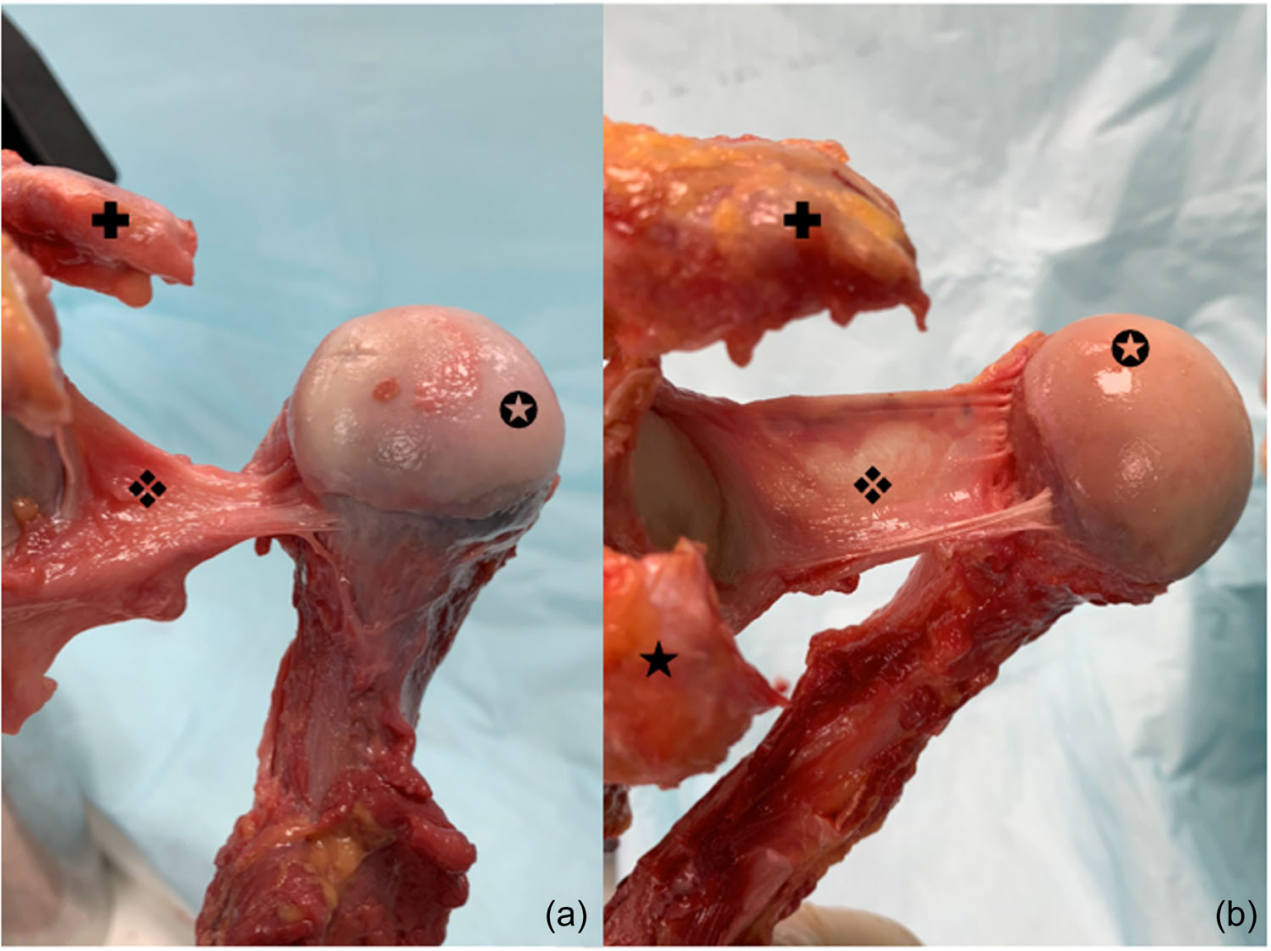
Two aspects of the posterior band of the inferior glenohumeral ligament (IGHL). ❖, posterior band of the IGHL; ✪, humeral head; ✚, acromion; ★, coracoid process. (a) Proximal insertion on the glenoid close to the labrum, from 6 to 9 h, with small and thick distal insertion. (b) Proximal insertion on the glenoid close to the labrum, from 6 to 9 h, with large and thin distal insertion.

In ER2, the AB tensioned at 81 ± 19° (30–100) of external rotation (always at 90° ABD). The external rotation necessary to tension AB was specimen‐dependent, and therefore had a large range. Anterior translation was limited by the AB. After sectioning the AB, anterior translation remained limited by the PB, which wrapped around the humeral head and locked the joint after an increase in external rotation. After sectioning the AB, external rotation increased until full tensioning of the PB. The PB wrapped around the humeral head and locked the joint. In this position, anterior translation was limited by the PB (Figure 2).

In the Hawkins–Kennedy test position, posterior translation was limited by the PB alone in four cases and by the PB associated with its posterosuperior capsular expansion in eight cases. After resection of this posterosuperior capsular expansion, the PB dislocated under the humeral head and did not stabilize the joint in the last eight cases.

There was no tensioning of the AB and PB, during forward and backward elevation of the humeral head with respect to the glenoid fossa, except for one case with tension of the PB at 130° forward elevation (FWD).

**Figure 3:**
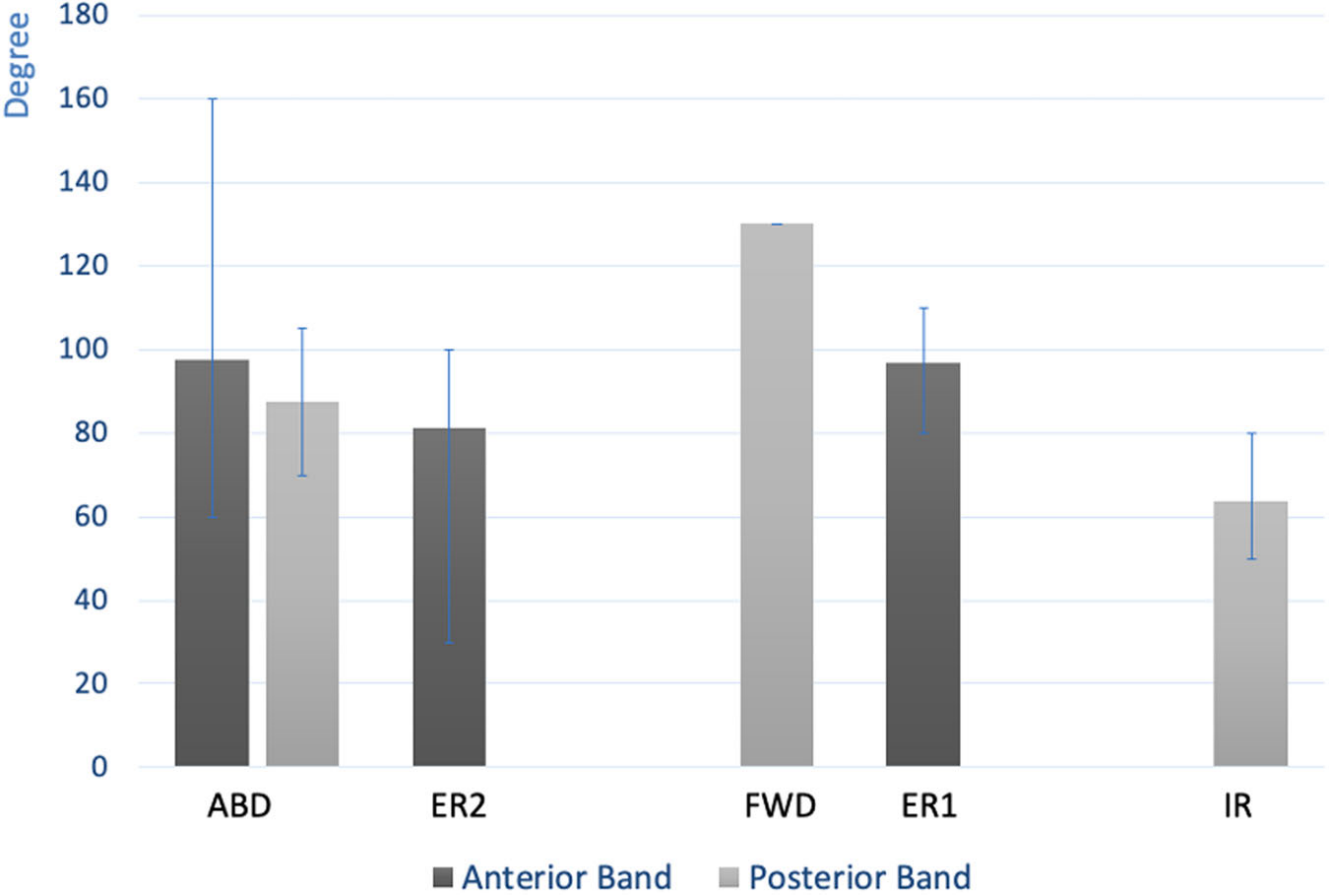
Degree of position of movement in which the anterior or posterior band is under maximum tension. Evaluation was performed for each position tested. ABD, abduction; ER1, external rotation 1; ER2, external rotation 2; FWD, forward elevation; IR, internal rotation.

Figure 3 summarizes the data according to the range of motion evaluated in degrees of mobility. It evaluates the tension of intact AB and PB. The AB and PB were both put under tension during abduction, but the PB is systematically in maximum tension before the AB. In ER2, the AB is the only one in tension, as soon as 80° of ER. To obtain a tension of the PB, it is necessary to remove the AB. During FWD elevation, only the AB is under tension, at a mean value of 130° of FWD elevation. In ER, only the AB is under tension, at a mean value of 95° pure ER. In IR, only the PB is put under tension, with maximum tension at 65° of IR.

## DISCUSSION

The current study of the PB of the IGHL describes the dynamic positions of the PB, in several positions of the shoulder and after removal of the AB. It could show a potentially high role of the PB in anterior instability and thus stabilization of the shoulder. It also assumes that posterior instability has more complex biomechanics than expected [14].

In a cadaveric study, Bigliani et al. [2] explored the tensile properties of the global inferior glenohumeral ligament. They divided the IGHL in three parts: anterosuperior, inferior and posteroinferior with the last segment probably containing the PB of the IGHL. Uramaya et al. [13] analysed the position and stabilizing function of AB and PB in the intact shoulder. The AB and axillary pouch showed significant strain increases when the arm was elevated and externally rotated in the coronal and scapular planes but no increase in the sagittal plane. The PB showed no strain in the coronal and scapular planes, but a significant strain increases with the arm elevated and internally rotated in the sagittal plane. Massimini et al. [8] evaluated the position of AB and PB of the IGHL. Their study was performed on healthy adults, using fluoroscopy, without seeing directly the insertional zone of each band. They found that the AB and axillary pouch showed strain increases when the arm was elevated and externally rotated in the coronal and scapular planes but no increase in the sagittal plane. The PB showed no strain in the coronal and scapular planes, but strain increases with the arm elevated and internally rotated in the sagittal plane. Itoigawa and colleagues presented the limited information we have on the PB of the IGHL and the need to better understand its anatomy and role in shoulder stability [4, 5]. It undoubtedly has an important role as Pouliard and colleagues [9, 11] demonstrated that it was not possible to have traumatic anterior instability of the shoulder without having both anterior and posterior lesions during glenohumeral dislocation. These authors found posterior lesions in 30% of cases. This structure is often difficult to identify, and in our study, it was confused with the posterior capsule in 2 of the 12 cases. It has insertions similar to those of the AB but its mean length and width are slightly larger than those of the AB while not being as thick.

**Figure 4:**
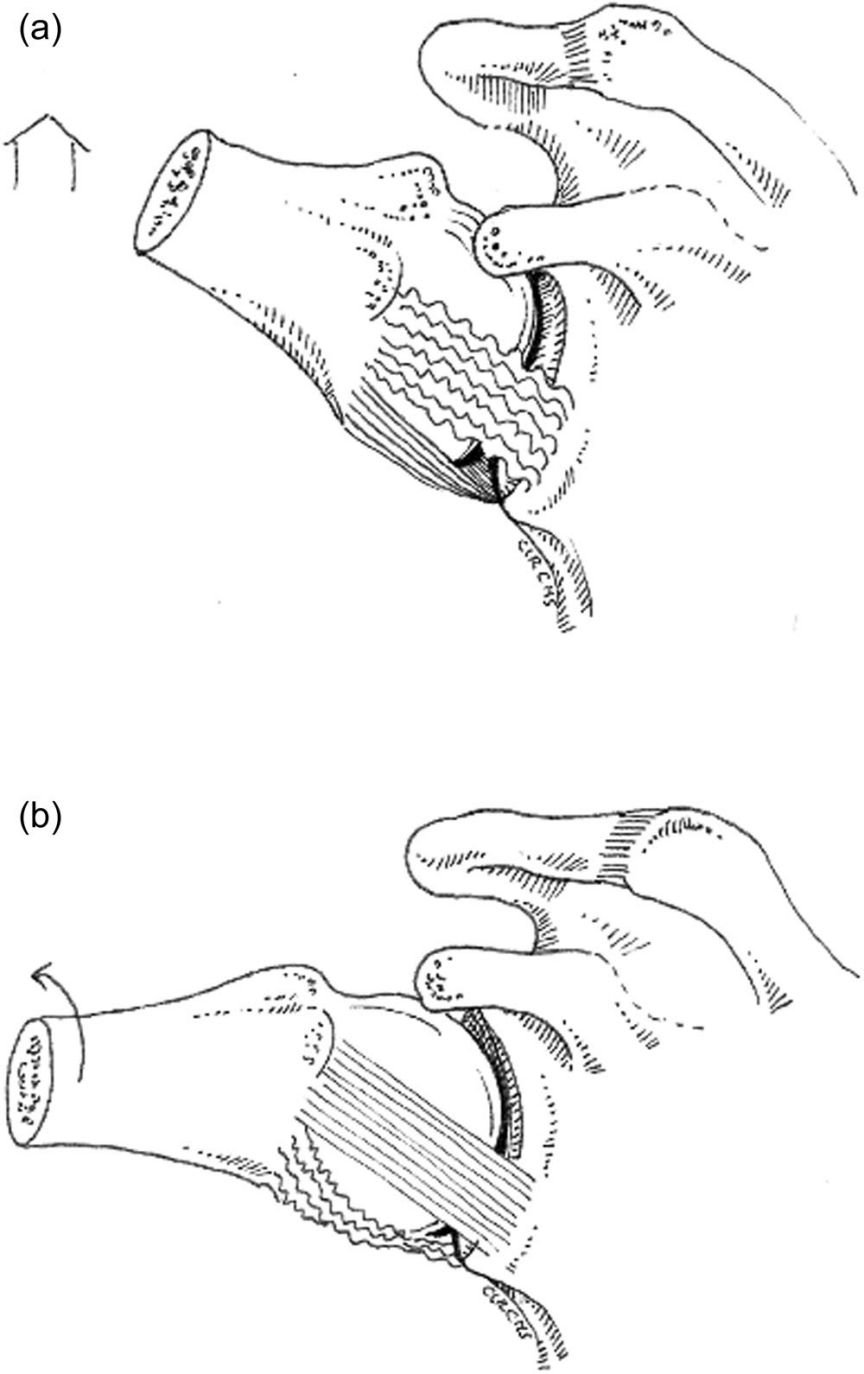
Schematic demonstrating the tensions of the anterior band (AB) and posterior band (PB) of the inferior glenohumeral ligament. (a) Pure abduction showing the AB is not under tension while the PB is under tension. (b) External rotation is needed with further abduction to lessen the tension of the PB.

The current study demonstrates that the AB and axillary pouch, which showed the greatest strain in ABD and external rotation, are anterior stabilizers, whereas the posterior band, which showed the greatest strain in flexion and IR, is a posterior stabilizer. It also shows that in a position of pure ABD, the PB may impose external rotation to increase the movement of the shoulder (Figure 4). The AB appears not to be involved in this movement.

This may play a role while testing for instability or ABD, such as the Gagey test. It is possible that this difference, defined by 20° more mobility by some authors, corresponds to a loss of the anatomical effect of ER2 imposed by the PB, and therefore a lesion to this band. In the case of a positive ABD test, the PB should be examined systematically.

In the current study, when the AB is sectioned, this increases ER2 in ABD, and the PB appears to potentially play a role in anterior stabilization of the shoulder. The movement necessary to tension AB in ER2 and ABD was specimen‐dependent, and therefore had a large range.

Conversely, if the PB is intact, it is not possible to dislocate the shoulder in ER1, as it is locked by the band wrapping forward around the head. It is therefore possible that dislocation in ABD external rotation could occur as a result of an isolated lesion to the AB, only if external rotation is not at a maximum. This difference in external rotation could be one of the explanations for the variable localization of the Hill–Sachs lesion. The greater the external rotation, the higher the lesion will be on the humeral head. This idea should be confirmed by other studies.

**Figure 5:**
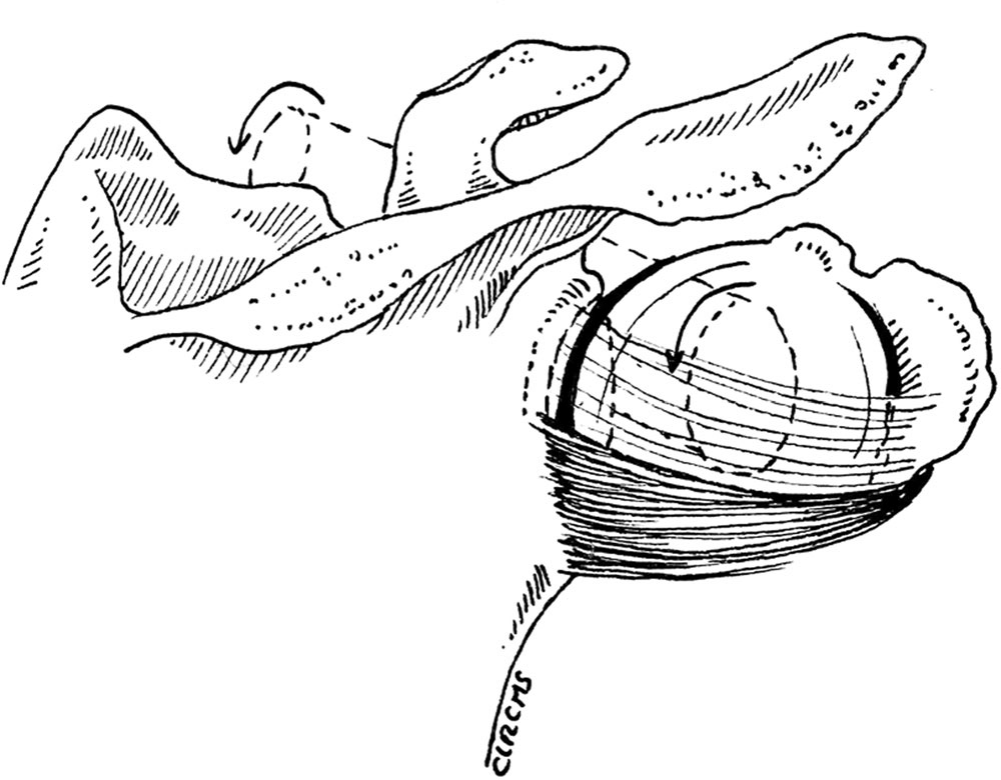
Schematic showing posterior structures during Hawkins–Kenedy test or Kim position. Forward elevation 90°, adduction, posterior compression: all posterior structures are involved in posterior stability.

This study seems to implicate the PB less as a dominant element in the mechanism of posterior instability. In contrast to the AB in anterior instability, the PB appears insufficient to prevent excessive posterior translation. By contrast, the posterior capsule appears to be a determining element as a suspensory and stabilizing element of the PB. When the posterior capsule was removed, the PB no longer had a stabilizing effect in the majority of cases in the Hawkins–Kennedy or Kim test, because the PB slides under the humeral head. When the posterosuperior capsule keeps the posteroinferior band of the ligament in place it is not possible to dislocate the shoulder backwards. These two structures therefore have a joint role in posterior instability (Figure 5).

This study has some limitations. It was an anatomical study with all that involves in terms of age of the patients and aging of the tissues. However, the shoulders were selected for the complete absence of degenerative lesions or previous surgery. The functional analysis was carried out on a dedicated support but without numerical measurements. Measurement of the angles was carried out using goniometers but we multiplied the measurements, as validated in many previous anatomical studies. Tension was visually estimated and not measured, so estimates of biomechanical function need to be confirmed by precision biomechanical testing.

In the same way, measurement of soft tissue can always be improved as it is influenced by the quality of the dissection. The bands could not always be identified easily. Measurement of the length, width and thickness depends on the glenohumeral position at the time of measurement and varies according to the stretch and laxity of the tissues. The current study is only a gross measurement of passive movements and not dynamic evaluation. Specimens used in this study were all cadavers, which are biomechanically a long way from the populations at risk of instability, patients with ligamentous laxity.

## CONCLUSION

When the IGHL is isolated, ligament limitation of glenohumeral ABD seems to be uniquely dependent on the PB. In the Hawkins and Kennedy position, the AB is the first constraint. In the case of an isolated lesion to the AB, the PB participates in anterior stabilization of the shoulder by wrapping around the humeral head that cannot dislocate. These findings confirm the role of the PB in glenohumeral joint stability.

## AUTHOR CONTRIBUTIONS

*Study conception and design*: Geoffroy Nourissat and Anselme Billaud. *Data collection*: Claire Bastard, Vincent Salabi and Claire Cammas. *Analysis and interpretation of results*: Claire Bastard and Geoffroy Nourissat. *Draft manuscript preparation*: Claire Camas and Geoffroy Nourissat. All authors reviewed the results and approved the final version of the manuscript.

## CONFLICT OF INTEREST STATEMENT

The authors declare no conflict of interest.

## ETHICS STATEMENT

The study was approved by the local ethics committee.
